# Mesoporous tantalum oxide nanomaterials induced cardiovascular endothelial cell apoptosis via mitochondrial-endoplasmic reticulum stress apoptotic pathway

**DOI:** 10.1080/10717544.2022.2147251

**Published:** 2022-12-19

**Authors:** Yuanyong Jiao, Xiwei Zhang, Hongyu Yang, Hao Ma, Junjie Zou

**Affiliations:** Department of Vascular Surgery, The First Affiliated Hospital with Nanjing Medical University (Jiangsu Province Hospital), Nanjing, China

**Keywords:** Tantalum oxide, HCMECs, apoptosis, mitochondria, endoplasmic reticulum, animal model

## Abstract

Along with its wide range of potential applications, human exposure to mesoporous tantalum oxide nanomaterials (PEG@mTa_2_O_5_) has substantially risen. Accumulative toxic investigations have shown the PEG@mTa_2_O_5_ intake and cardiovascular diseases (CVD). Endothelial cell death is crucial in the onset and development of atherosclerosis. Still, the molecular mechanism connecting PEG@mTa_2_O_5_ and endothelium apoptosis remains unclear. Herein, we studied the absorption and toxic action of mesoporous tantalum oxide (mTa_2_O_5_) nanomaterials with polyethylene glycol (PEG) utilizing human cardio microvascular endothelial cells (HCMECs). We also showed that PEG@mTa_2_O_5_ promoted apoptosis in endothelial cells using flow cytometry and AO-EB staining. In conjunction with the ultrastructure modifications, PEG@mTa_2_O_5_ prompted mitochondrial ROS production, cytosolic Ca^2+^ overload, ΔΨm collapse, and ER stress verified by elevated ER-Tracker staining, upregulated XBP1 and GRP78/BiP splicing. Remarkably, the systemic toxicity and blood compatibility profile of PEG@mTa_2_O_5_ can greatly improve successive therapeutic outcomes of NMs while reducing their adverse side effects. Overall, our findings suggested that PEG@mTa_2_O_5_-induced endothelium apoptosis was partially mediated by the activation of the endoplasmic reticulum stress-mitochondrial cascade.

## 1. Introduction

One dimension of a nanomaterial must be 100 nm or less. The potential uses of nanomaterials in biological domains are growing rapidly with the advancement of nanotechnology (Niculescu, 2020). Gold nanomaterials for biodiagnosis, magnetic nanomaterials for bioimaging and drug administration, and mesoporous silica nanomaterials (MSNs) for drug delivery are just a few examples of nanomaterials (NMs) that have garnered attention recently for their potential biomedical uses (Ghaferi et al., 2021). However, growing worries about the potential nanotoxicity of materials used in various biological applications have been voiced worldwide (Croissant et al., 2018). More nanomaterials, including high-Z elements, including Hf, Ta, W, Bi, Au, and rare earth metals, have been investigated as sensitizers to boost the efficacy of radiotherapy in treating tumors due to the Auger effect (Kesse et al., 2019). In this study, a novel kind of mesoporous tantalum oxide (mTa_2_O_5_) nanomaterials are constructed as a dual-functional nano-DDS for improved chemo/radiotherapy, taking inspiration from the formation of mesoporous silica to facilitate effective drug loading (Kashfi-Sadabad et al., 2019; Shirazi-Amin et al., 2020; Koshevaya et al., 2021). The polyethylene glycol (PEG) is incorporated into the mTa_2_O_5_ nanomaterials using a layer-by-layer polymeric coating process after being synthesized using a simplified one-step soft template method (Nagrath et al., 2021).

The biocompatibility of tantalum oxide nanomaterials (Ta_2_O_5_-NMs) has recently increased interest as a CT contrast agent (Pasupulety et al., 2022). Several research groups reported that water-soluble tantalum oxide nanomaterials (Ta_2_O_5_-NMs, sub-10 nm) for X-ray imaging since the X-ray reduction coefficient of Ta is more cost-effective than gold nanomaterials. However, rapid renal clearance of these NMs prevented their widespread biouse for computed tomography (CT) fluorescence imaging (Bhaduri et al., 2022). Oh, and coworkers fabricated uniform-sized TaOx-NMs using a microemulsion approach. They adapted the nanomaterials using PEG-silane and RITC- for CT fluorescence imaging (Kashfi-Sadabad et al., 2019). Multifunctional Fe_3_O_4_/TaOx core/shell NMs were constructed by Lee and coworkers reported for X-ray CT-MR imaging, and TaOx@PPy NMs were designed for CT/photoacoustic imaging-guided photothermal cancer treatment (Koshevaya et al., 2021). Still, no report has been made using TaOx-NMs as a drug carrier to load chemotherapeutic medicines for imaging-guided targeted drug delivery and controlled release (Bhaduri et al., 2022).

Exposure to NMs may aggravate cardiovascular illness and may disturb cardiovascular homeostasis in healthy persons, according to evidence from epidemiological studies (Chen et al., 2020). Application areas include injecting NMs as nanodiagnostics, nanomedicines, or implant-derived detritus, all of which would result in fast NMs circulation within the circulatory system. NP-endothelial interactions may be harmful because in-blood NMs direct contact with vascular endothelial cells (VECs), located around the blood vessels’ lumen, creating a barrier between blood and tissues (Zahedi et al., 2021). Rodents showed that MSNs (<500 nm in size) might pass the blood-brain barrier following intranasal instillation, providing evidence that these NMs could have an inevitable effect on the vascular endothelium (Chan et al., 2019). Before any MSNs are pronounced safe for widespread usage, extensive study of their interactions with VECs is essential. Human cardiac microvascular endothelial cells (HCMECs) were shown to have mitochondrial dysfunction produced by MSNs and membrane damage. Also, MSNs dramatically enhanced the toxicity of human aortal endothelial cells (HAECs) in a concentration-dependent manner, suggesting that MSNs’ potential uses are constrained by the increased cardiovascular risk they provide (Cao, 2018).

As well as vascular endothelial cells, HCMECs are commonly utilized in investigating nanotoxicity because of the characteristic endothelial phenotype (Feng et al., 2019). Here, we analyzed PEG@mTa_2_O_5_ uptake and apoptotic induction in HCMECs and showed potential molecular pathways of PEG@mTa_2_O_5_-induced endothelial apoptosis. The data showed that ingested PEG@mTa_2_O_5_ might induce endothelium apoptosis via ER stress-mitochondria cascade-mediated signaling and oxidative damage. The systemic toxicity and blood compatibility profile of PEG@mTa_2_O_5_ can greatly improve successive therapeutic outcomes of NMs while reducing their adverse side effects. Our results not only fill in the gaps in knowledge about PEG@mTa_2_O_5_-induced endothelium toxicity but also shed light on the theoretical underpinnings of preventing and treating CVD.

## 2. Material and methods

### 2.1. Materials and reagents

NH_2_-mPEG (Mw-5,000) was acquired from Shanghai Ponsure Biotechnology Inc. (Shanghai, China). Tantalum ethoxide was purchased from Changzhou Beiyuanxin Biotechnology Co., Ltd. (Changzhou, China). 3-aminopropyltrimethoxysilane (APTMS), ammonium hydroxide (NH_3_. H_2_O), N-(3-dimethylaminopropyl-N′-ethylcarbodiimide) hydrochloride (EDC) were obtained from Sinopharm (Shanghai Sinopharm Group Chemical Reagent Co., Ltd.). Ultrapure water (18.2 MΩ cm^−1^) applied in all experiments was produced by a Milli-Q purification system. Dulbecco’s modified Eagle’s medium (DMEM), fetal bovine serum (FBS), penicillin − streptomycin solution, and trypsin were acquired from Life Technologies (Shanghai, China). Thiazolyl blue tetrazolium with purity above 98.0% was purchased from Sigma-Aldrich Co., Ltd. (Shanghai, China). ER-Tracker Red/MitoTracker Green, Annexin V-FITC, and propidium iodide were obtained from Beijing Solarbio Science & Technology Co., Ltd. JC-1 for ΔΨm by Solarbio Technology Co., Ltd. (Beijing, China). Fluo-3 AM (an intracellular calcium indicator) was obtained from Maokangbio (China).

### 2.2. Fabrication of mesoporous tantalum oxide nanomaterials (mTa_2_O_5_)

For the fabrication of mTa_2_O_5_ nanomaterials, CTAB (640 mg) and EtOH (3.2 mL) were mixed with an aqueous solution (40 mL). At room temperature, stirring continuously, EtOH (7.2 mL) containing tantalum ethoxide (120 µL) was added. After 3-h continuous stirring, the fabricated mTa_2_O_5_ nanomaterials were centrifuged (15,000 rpm) and rinsed with distilled water thrice to eliminate excess CTAB. The obtained nanomaterials were ultrasonically distributed in EtOH solution.

### 2.3. Surface modification of mTa_2_O_5_ nanomaterials

To the mTa_2_O_5_-ethanol solution, we added the first APTMS (350 µL) and NH3 • H2O (50 µL). The solutions were heated at 80 °C while continuously agitated for a whole night. After being exposed to ethanol, the resulting nanomaterials were diluted in 40 mL of diiodomethane. The second step was gradually adding the solution to PAA (Mw = 1,800, 5 mg/mL) that was being sonicated simultaneously. The mTa_2_O_5_/PAA solution was centrifuged (14,800 rpm) to remove impurities before being re-dissolved in water. This process took 2 h. The conjugation between carboxyl and amino groups was induced by adding EDC (30 mg) to the solution after lowering the pH to 7.5. Finally, after 30 min of ultrasonication, EDC (35 mg) was added to a mixture of mTa_2_O_5_/PAA and mPEG-5K-NH_2_ (250 mg) solution. The resulting PEG@mTa_2_O_5_ nanomaterials were filtered by ultrafiltration filters and then rinsed three times with an aqueous solution after being agitated overnight. After obtaining PEG@mTa_2_O_5_ nanomaterials, they were re-distributed in aqueous solutions and kept at −4 °C for further use (Chen et al., 2017).

### 2.4. Characterization of PEG@mTa_2_O_5_

Transmission electron microscopy (TEM) was accomplished with a Hitachi H-7500 instrument operated at an acceleration voltage of 40-120 kV (100 V/setting). The Zeta potential of the materials was measured on a Zetasizer Nano (Malvern Instruments). The absorbance was calculated at 570 and 630 nm using a microplate reader (Thermo Fisher Scientific, Waltham, MA). Specific surface area and corresponding pore-size distribution of nanomaterials were determined by a Micromeritics Tristar 3000 system (Micromeritics, Norcross, GA, USA) using the nitrogen (N2) adsorption-desorption isothermal technique. Cell images were taken using a fluorescence microscope (Olympus CKX53, Japan). Nanomaterials were centrifuged (30,000 × g) to remove unencapsulated components, followed by redispersion in Milli-Q water by sonication before the investigations.

### 2.5. Cell culture and MTT assay

Human cardiac microvascular endothelial cell line (HCMECs) cells were acquired from Procell Life Science & Technology Co. Ltd. (Wuhan, China). The cells were cultured in Dulbecco’s modified Eagle’s medium DMEM) containing 10% of fetal bovine serum (FBS), 100 U/ml antibiotics (penicillin/streptomycin) and 0.05 mM β-mercaptoethanol. The HCMECs cells were cultivated in a humidified 5% CO_2_ atmosphere at 37 °C (Zhang et al., 2018; Del Turco et al., 2019; Wen et al., 2019).

The cytotoxicity of PEG@mTa_2_O_5_ was examined by a standard methyl thiazolyl tetrazolium (MTT) assay. Briefly, cells were seeded into 96-well plates at the cell density of 3,000 cells per well and cultured for 24-h. Then, the medium in all wells was replaced with fresh ones containing different concentrations of the nanomaterials (50, 100, 150, and 200 μg/mL), and the cells were cultured for another 24-h and 48-h. After that, MTT (0.5 mg/mL) was added to the culture medium in each well and incubated for an additional 4 h. Finally, each well was washed with sterile PBS thrice, and the medium was replaced with 150 μL DMSO to dissolve the residues. Three independent experiments were conducted for each concentration, and four replicates were performed in each independent investigation.

### 2.6. TEM observation

HCMECs were seeded under standard conditions for 24-h (conditions like those used for fluorescence experiments). Cell culture media was supplemented with 50 μg/mL PEG@mTa_2_O_5_ and incubated for 2-h at 37 °C. The HCMECs were then fixed for 45 min at RT in a mixture of paraformaldehyde, glutaraldehyde, and phosphate buffer. The cells were dehydrated using a graduated ethanol series before implanted in EPON resin. The resin block was then divided into ultrathin slices (100 nm thick) and dyed with 2% uranyl acetate for greater contrast imaging with the HR-TEM microscope.

### 2.7. Apoptosis measurement

For flow cytometry analysis, HCMECs were seeded in a 6-well plate (2 × 10^5^ cells in 2 ml of 1,640 medium per well). After incubation for 24-h, the culture medium was removed, and the fresh medium containing PEG@mTa_2_O_5_ (50, 100, 150, and 200 μg/mL) was added. After co-incubation with the nanomaterials for 4-h, the cells were washed thrice by PBS, collected by centrifugation, resuspended in 200 μl of PBS, and analyzed by flow cytometry (SYSMEX, Japan). The cells (1 × 10^6^ cells per well) were cultured in 6-well plates for 24-h. The cells were then exposed to PEG@mTa_2_O_5_ (50, 100, 150, and 200 μg/mL) for 24-h, respectively. Then, the cells were stained with AO-EB staining according to the manufacturer’s instructions (Shen et al., 2011).

### 2.8. Mitochondrial ROS assessment

Intracellular ROS generation in HCMECs was detected by MitoTracker Green and MitoSOX^TM^ Red, which could be oxidized to produce a fluorescent compound of MitoTracker Green in the presence of ROS. The medium was then replaced with fresh medium containing PEG@mTa_2_O_5_ (50, 100, 150, and 200 μg/mL) for humidified 5% CO_2_ atmosphere at 37 °C. After another 4-h of co-incubation, the medium was removed. Then, a fresh DMEM medium (1 mL) containing MitoTracker Green and MitoSOX^TM^ Red (20 μM) was added, followed by further incubation for 20 min (Jangid et al., 2021).

### 2.9. Δψm (MMP) determination

HCMECs were incubated with PEG@mTa_2_O_5_ (50, 100, 150, and 200 μg/mL) in DMEM for 4 h. The cells were washed twice with PBS and stained with (5, 5′, 6, 6′-tetrachloro-1,1′,3,3′-tetraethylbenzimi-dazolylcarbocyanineiodide) JC-1 (5 µg/mL) for 15 min, and the green fluorescence (the monomeric JC-1) and red fluorescence (the aggregated JC-1) were observed by using a microscope (Khatua et al., 2020).

The changes in MMP of HCMECs were evaluated using a cationic fluorophore with JC-1 dye. JC-1 is the dye especially used as a mitochondrial tracker. HCMECs exposed to PEG@mTa_2_O_5_ (50, 100, 150, and 200 μg/mL) were washed and suspended in PBS. The suspension was mixed with 2 μM of JC-1 dye and kept at 4 °C for 15 min. The total suspensions were then rinsed thrice with PBS. After incubation, the HCMECs were centrifuged with 1,500 g for 3 min, and the supernatant content with unbound dye was removed. This was then produced for fluorescence intensity analysis by flow cytometer.

### 2.10. Intracellular calcium level determination

Labeling the cells with the Fluo-3-AM calcium indicator could determine the amount of Ca^2+^ inside the cells. Briefly, HCMECs were treated with the Fluo-3-AM (4 μM for 15 min) following PEG@mTa_2_O_5_ treatment for 24-h. The last step was LSCM imaging of the cells. Meanwhile, FCM was used to assess and quantify the fluorescence intensities (Matsuo et al., 2007).

### 2.11. ER staining

For ER staining detection, 1 × 10^4^ HCMECs cells were incubated in 6-well plates overnight for attachment and then treated with ER-Tracker Red for 24-h. After the incubation with ER-Tracker Red (4 μM for 10 min), the HCMECs were washed twice with PBS. The last step was LSCM imaging of the cells.

Image analysis for ER staining was achieved by seeding cells on a cover-slip loaded six-well plate at 1 × 10^5^ cells/mL. 16-h after plating, cells were treated with PEG@mTa_2_O_5_. 12-h after, the ER-Tracker Blue-White DPX probe was added to the cells and incubated for 30 min under humidified 5% CO_2_ atmosphere at 37 °C. The loading solution was removed, and cells were then washed with PBS.

### 2.12. Quantitative real-time PCR analysis

Transcriptional level of GRP78/BiP evaluation was performed using the real-time reverse transcriptase-polymerase chain reaction (RT-qPCR). HCMECs (2 × 10^5^ cells/mL) were harvested after being treated as described above in the MTT assay. The total RNA was isolated from the cells using the Trizol reagent (Invitrogen, Carlsbad, CA, USA). Reverse transcription was treated with total RNA (1 μg) and each cDNA (1 μL) from each sample in a total volume of 20 μL using an M-MLV reverse transcriptase kit. PCR cycling conditions were: 40 cycles of 94 °C for 1 min, 60 °C for 1 min, and 72 °C for 2 min. The primers used for PCR were the same as in our previous work.

### 2.13. Western blot analysis

HCMECs (3 × 10^5^ cells/mL) were seeded into 6-well plates and cultured overnight. The cells were treated with PEG@mTa_2_O_5_ (50, 100, 150, and 200 μg/mL). SDS-PAGE separated the protein, and the relevant proteins were electrophoretically transferred onto a PVDF membrane (Immobilon P, Millipore). After blocking with 5% nonfat dry milk for 1 h, the PVDF membranes were incubated with the primary antibodies (Abcam, Cambridge, UK), including Caspase-12, Caspase-9, Caspase-3, Bcl-2, Bax, phospho-JNK, C-Jun N-terminal kinase (JNK), CHOP, IRE1α, and cytochrome c and with horseradish peroxidase-conjugated secondary antibodies for 1-h at 37 °C. GAPDH served as a protein loading control. The specific proteins were imaged using an enhanced chemiluminescence imaging system (Tanon 5200, China).

### 2.14. Biosafety evaluation

Male BALB/C nude mice were housed in the Laboratory Animal Center of The First Affiliated Hospital with Nanjing Medical University (Jiangsu Province Hospital), Nanjing 210029, China. All the animal studies were conducted following the principles of the Institutional Animal Care and Use Committee. The mice were randomly divided into five groups (*n* = 5/group), receiving tail vein injections of 200 μL saline and PEG@mTa_2_O_5_ (50, 100, 150, and 200 μg/mL). The body weights of the mice were monitored and recorded every 2 days. On the last day, the mice were sacrificed, and their organs were weighed. The major organs (i.e. liver, heart, kidney, spleen, and lung) were excised on day 9 and subsequently subjected to H&E staining for analyzing acute and chronic toxicity induced by the drugs.

For blood analysis, healthy ICR mice have intravenously injected with 200 μL saline and PEG@mTa_2_O_5_ (50, 100, 150, and 200 μg/mL) solution. Blood samples (*n* = 5/group) were collected on day 9, and hepatorenal parameters, including aspartate aminotransferase (AST), alanine aminotransferase (ALT), total bilirubin (TBIL), alkaline phosphatase (ALP), creatinine (Cr) and blood urea nitrogen (BUN), were analyzed.

## 3. Results

### 3.1. The characterization of PEG@mTa_2_O_5_

**Figure 1.**
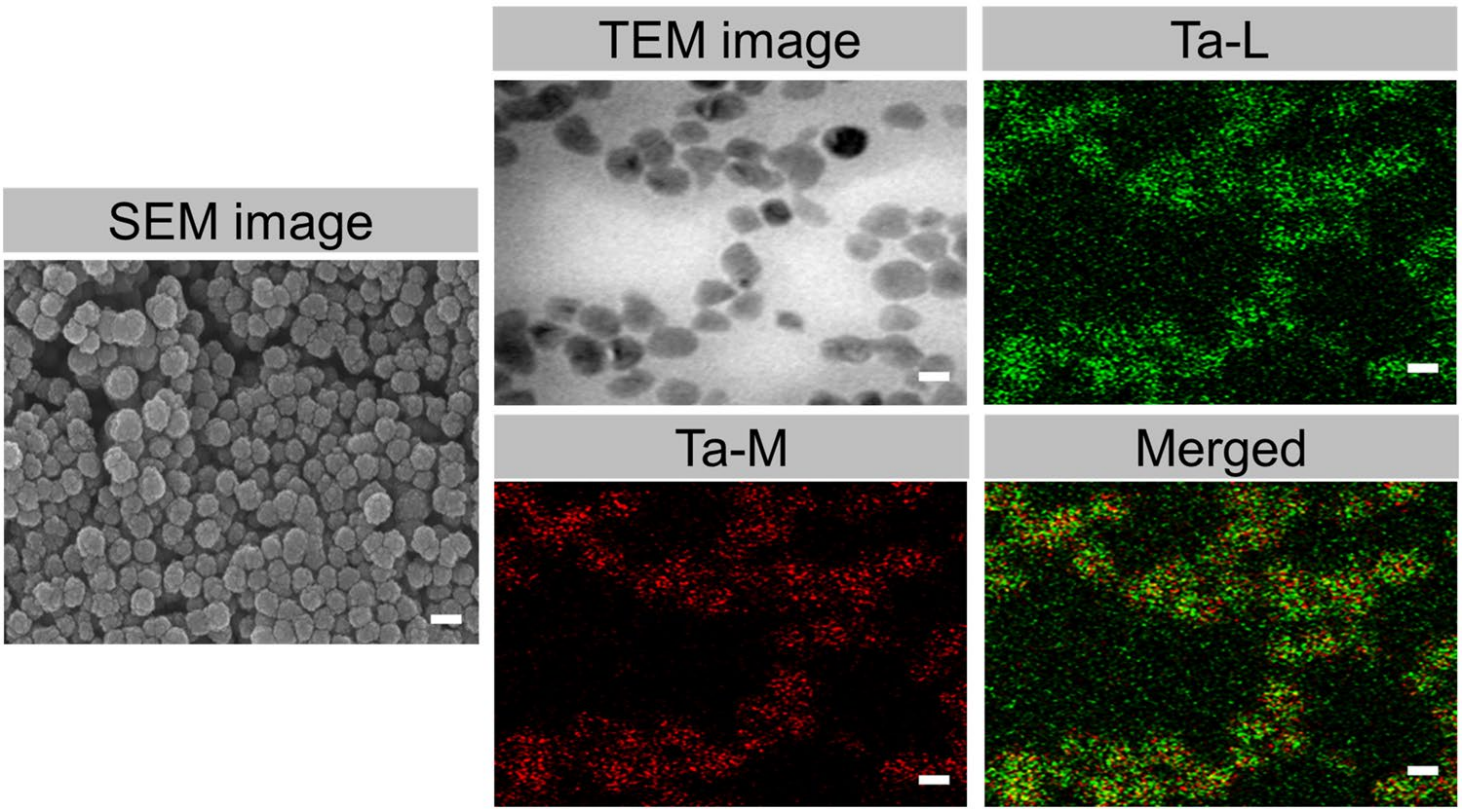
Morphology of PEG@mTa_2_O_5_ nanomaterials. SEM and TEM images of PEG@mTa_2_O_5_ nanomaterials. Scale bar 100 nm.

**Figure 2.**
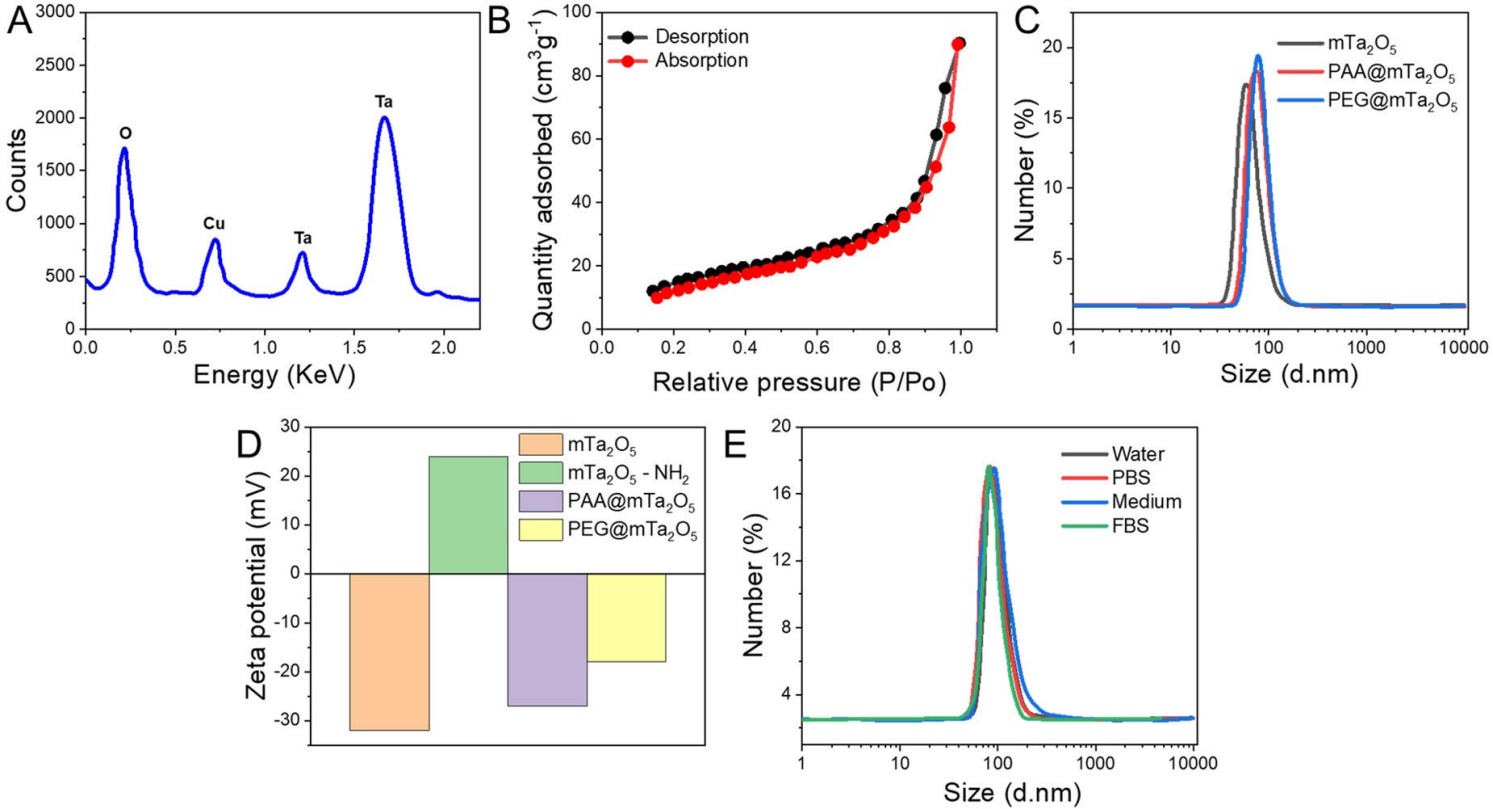
Characterization of PEG@mTa_2_O_5_ nanomaterials. (A) Energy dispersive (EDX) analysis. (B) N2 adsorption/desorption isotherms of mTa_2_O_5_ NMs. (C) DLS measurements of mTa_2_O_5_, PAA@mTa_2_O_5_, and PEG@mTa_2_O_5_. (D) Zeta potential changes of mTa_2_O_5_, mTa_2_O_5_-NH_2_, PAA@mTa_2_O_5_, and PEG@mTa_2_O_5_ dispersed aqueous solution. (E) Size distribution of mTa2O5-PEG dispersed in various solutions (water, FBS, cell culture medium, and PBS) after incubation for 24 h.

In our study, mTa_2_O_5_ nanomaterials were fabricated using a sol-gel surfactant-templating process. The mesoporous spherical form of mTa_2_O_5_ NMs could be readily detected under the SEM and TEM images (Figure 1). The elemental mapping and EDX spectral analysis further proved the formation of mTa_2_O_5_-NMs (Figure 1). XPS analysis demonstrated that mTa_2_O_5_ NMs were constructed of pentavalent tantalum (Figure 2A). The XRD spectra of as-synthesized mTa_2_O_5_ NMs confirmed their amorphous structure (Figure 2A). N2 adsorption-desorption isotherm curve was used to determine the average pore sizes of mTa_2_O_5_ NMs to be 4.3 nm and the specific surface area to be 90.1 m^2^ g ^− 1^ (Figure 2B). Despite the high density of α-mTa_2_O_5_ (8.38 g cm^−3^), the surface morphology of those mTa_2_O_5_ NMs is relatively considerable.

Their surfaces were modified to construct mTa_2_O_5_ NMs more soluble in water and stable in physiological fluids before they were used in bio-applications. By interacting with aminopropyltriethoxysilane, mTa_2_O_5_ NMs in their as-made form were covalently changed with amino groups. The NMs were subsequently conjugated with mPEG-NH_2_ (Mw = 5,000) by creating amide bonds after being covered with anion exchange PAA through electrostatic interactions. Zeta potential and dynamic light scattering (DLS) studies confirmed the sequential transformation processes (Figure 2D). Nanomaterials surface-modified with PEG showed no discernible morphological change (Figure 2D). The PEGylated mTa_2_O_5_ NMs had a hydrodynamic size of ∼90 nm (Figure 2C). They were stable in FBS, cell culture medium, PBS, and aqueous solution for 72-h without forming a precipitate or experiencing significant hydrodynamic size changes (Figure 2E).

### 3.2. Cytotoxicity of PEG@mTa_2_O_5_

After 24-h of interaction with PEG@mTa_2_O_5_, cellular morphology and cell viability were assessed to assess any potential harm to endothelial cells. Cell-density decrease, irregular shape, and cellular shrinkage were the hallmarks of the morphological alterations in HUVECs as the PEG@mTa_2_O_5_ dose increased. The vitality of the cells was directly proportional to the degree to which their shape had changed. Cell viability was dramatically reduced when cells were exposed to PEG@mTa_2_O_5_ at 50, 100, 150, and 200 μg/mL, falling to 92.93%, 81.24%, 42.68%, and 33.44%, respectively, compared to that in the control group. Cell viability decreased with time, indicating that PEG@mTa_2_O_5_ toxicity to HUVECs was both times- and dose-dependent. Furthermore, 24-h incubation of HUVECs with PEG@mTa_2_O_5_ developed a statistically substantial increase in LDH release assessed to the control. Cell viability and LDH-leakage showed significant agreement, demonstrating that PEG@mTa_2_O_5_ caused harm to the cell membrane.

### 3.3. The uptake of PEG@mTa_2_O_5_

**Figure 3.**
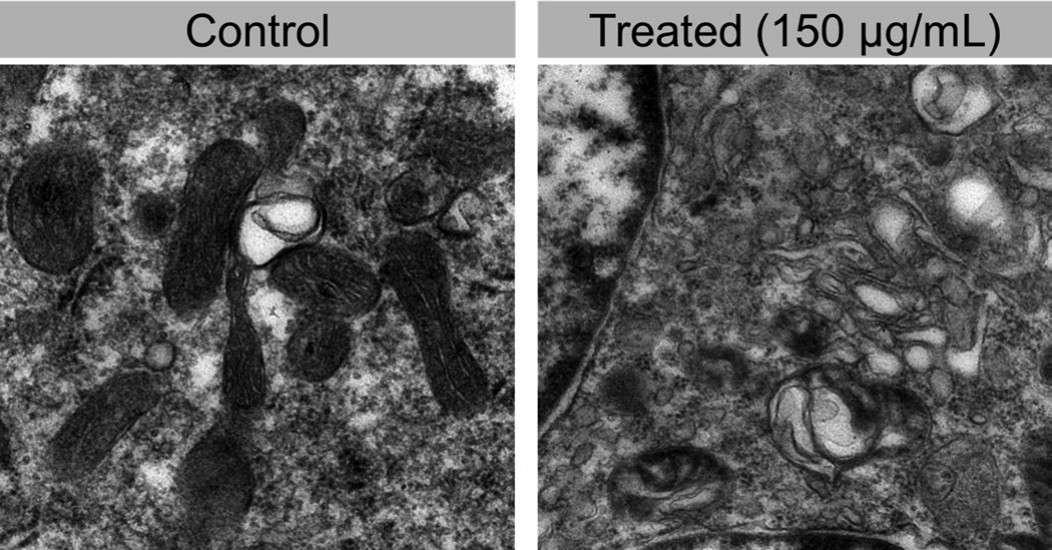
Uptake of the PEG@mTa_2_O_5_ by HCMECs. Cells were exposed to 150 µg/mL PEG@mTa_2_O_5_ for 24 h. The presence inside HCMECs of the NMs was examined by TEM analysis. Scale bar 200 µm.

Human cardiac microvascular endothelial cells (HCMECs) were treated with these nanomaterials’ uptake with PEG@mTa_2_O_5_ (Figure 3). Subsequent transmission electron microscopy (TEM) analysis revealed that PEG@mTa_2_O_5_ had been deposited in the cytoplasm, namely in the mitochondrial and endoplasmic reticulum (ER) regions in both agglomerates and isolated particles. Autophagic vacuoles, some of which encircled PEG@mTa_2_O_5_ and included remains of degraded cytoplasmic components, were also readily detected in PEG@mTa_2_O_5_-treated endothelial cells. In addition, aggregated ER and bloated mitochondria with damaged cristae were noticed.

### 3.4. Apoptosis induced by PEG@mTa_2_O_5_

**Figure 4.**
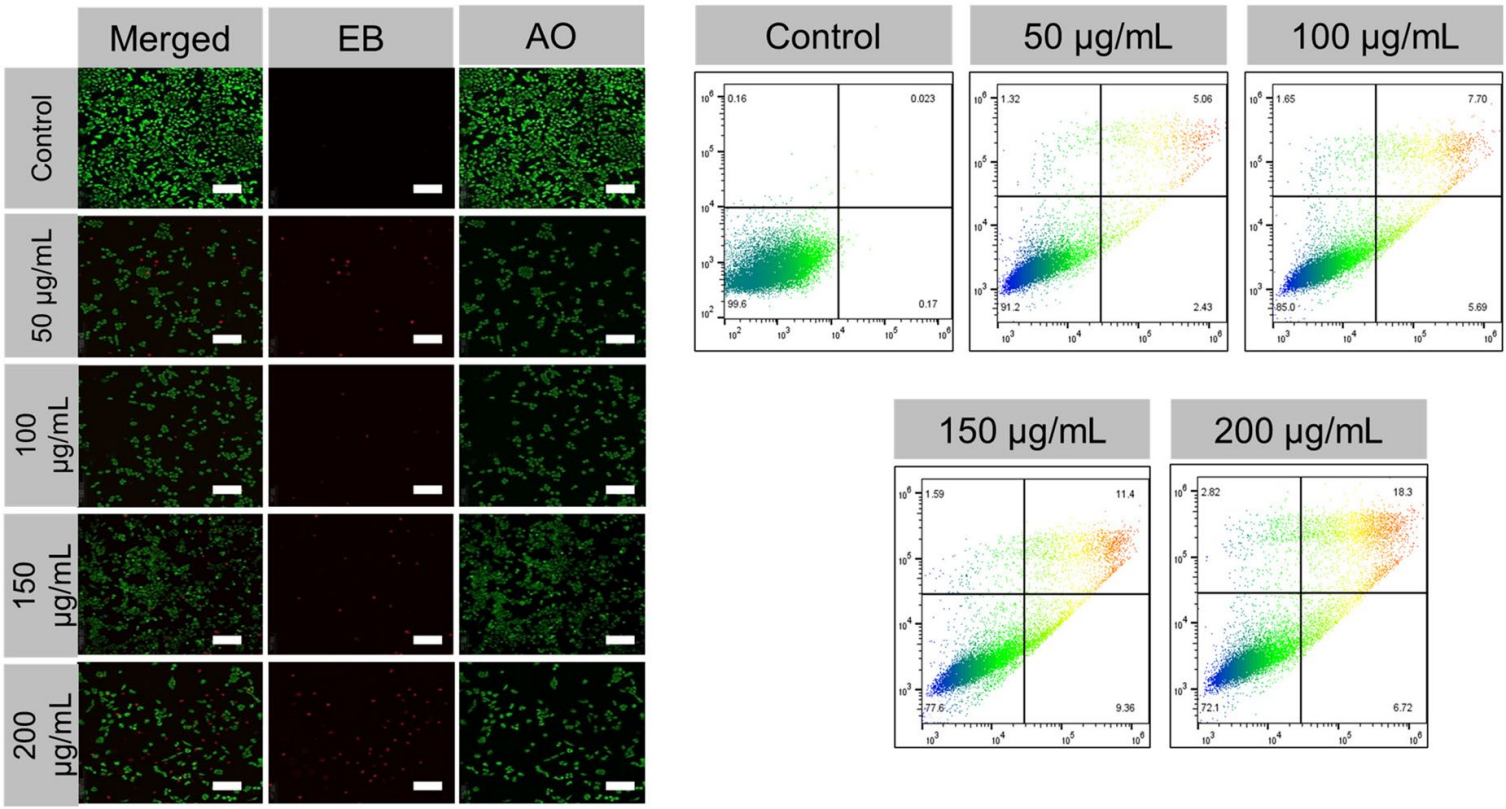
Apoptosis induction by PEG@mTa_2_O_5_ by HCMECs. After PEG@mTa_2_O_5_ treatment with different concentrations for 24 h, HCMECs were stained with acridine orange (AO)-ethidium bromide (EB). Scale bar 20 µm. The flow cytometry analysis of PEG@mTa_2_O_5_ treatment with different concentrations for 24 h, cells stained with Annexin V-FITC/PI.

The HCMECs were treated with PEG@mTa_2_O_5_ (50, 100, 150, and 200 μg/mL) for 24 h and stained with AO-EB staining to examine whether the cell death was due to apoptosis. The untreated HCMECs yielded about 98.5% viable cells. The AO (green fluorescence) was evenly distributed in these HCMECs, with normal nuclear morphology and negative red fluorescence (Figure 4). In all the treated samples, the proportion of viable cells decreased significantly. In contrast, as evidenced by the number of reddish-orange fluorescence-colored cells, the proportion of apoptotic cells increased dramatically in all treatments (Figure 4). At the 200 μg/mL concentration, PEG@mTa_2_O_5_ caused the highest percentage of apoptotic cells in HCMECs, demonstrating that PEG@mTa_2_O_5_ induced the highest rate of apoptotic cells (Figure 4). After 24-h treatment with HCMECs, the detection of apoptotic staining with Annexin V/PI was investigated using a flow cytometer (FCM). Further, the apoptosis quantification data obtained using FCM assay demonstrated a dose-response capacity of apoptosis triggering by PEG@mTa_2_O_5_ (Figure 4).

### 3.5. Mitochondrial ROS triggered by PEG@mTa_2_O_5_

**Figure 5.**
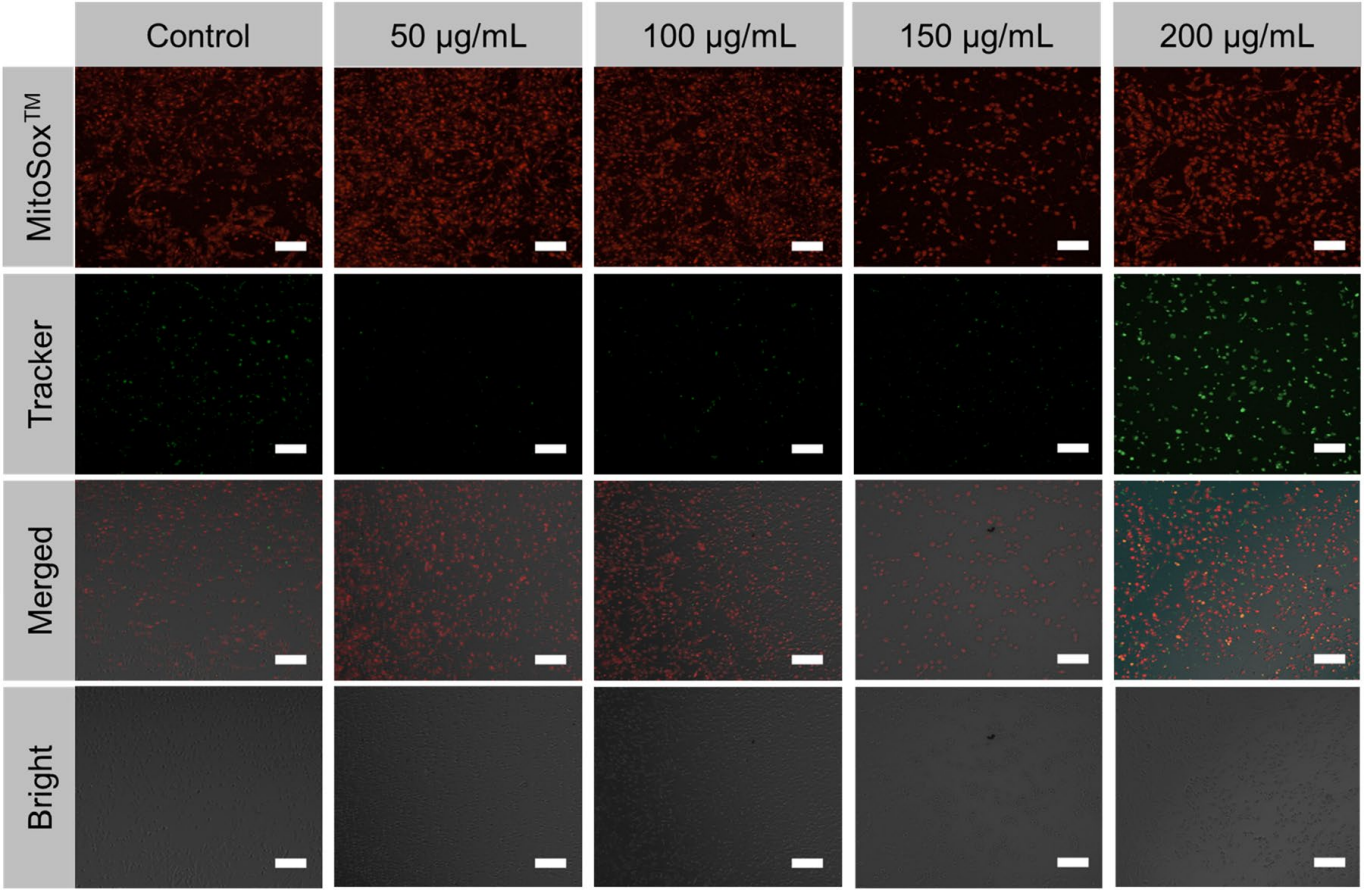
Mitochondrial oxidative stress triggered PEG@mTa_2_O_5_ by HCMECs. The images of HCMECs are stained with MitoTracker Green and MitoSOX™ fluorescence. Scale bar 100 µm.

The predominance of reactive oxygen species (ROS), a recognized inducer of apoptosis, is produced by mitochondria. Under LSCM, we measured mitochondrial ROS levels using MitoTracker Green and MitoSOX^TM^ Red, mitochondria-specific probes. Endothelial cells treated to PEG@mTa_2_O_5_ fluoresced more MitoSOX^TM^ Red, as shown in Figure 5. Mitochondrial reactive oxygen species (ROS) were reflected by the MitoSOX^TM^ Red/MitoTracker Green ratio. Consequently, PEG@mTa_2_O_5_ exposure increased the generation of ROS in mitochondrial (Figure 5).

### 3.6. Δψm collapse induced by PEG@mTa_2_O_5_

**Figure 6.**
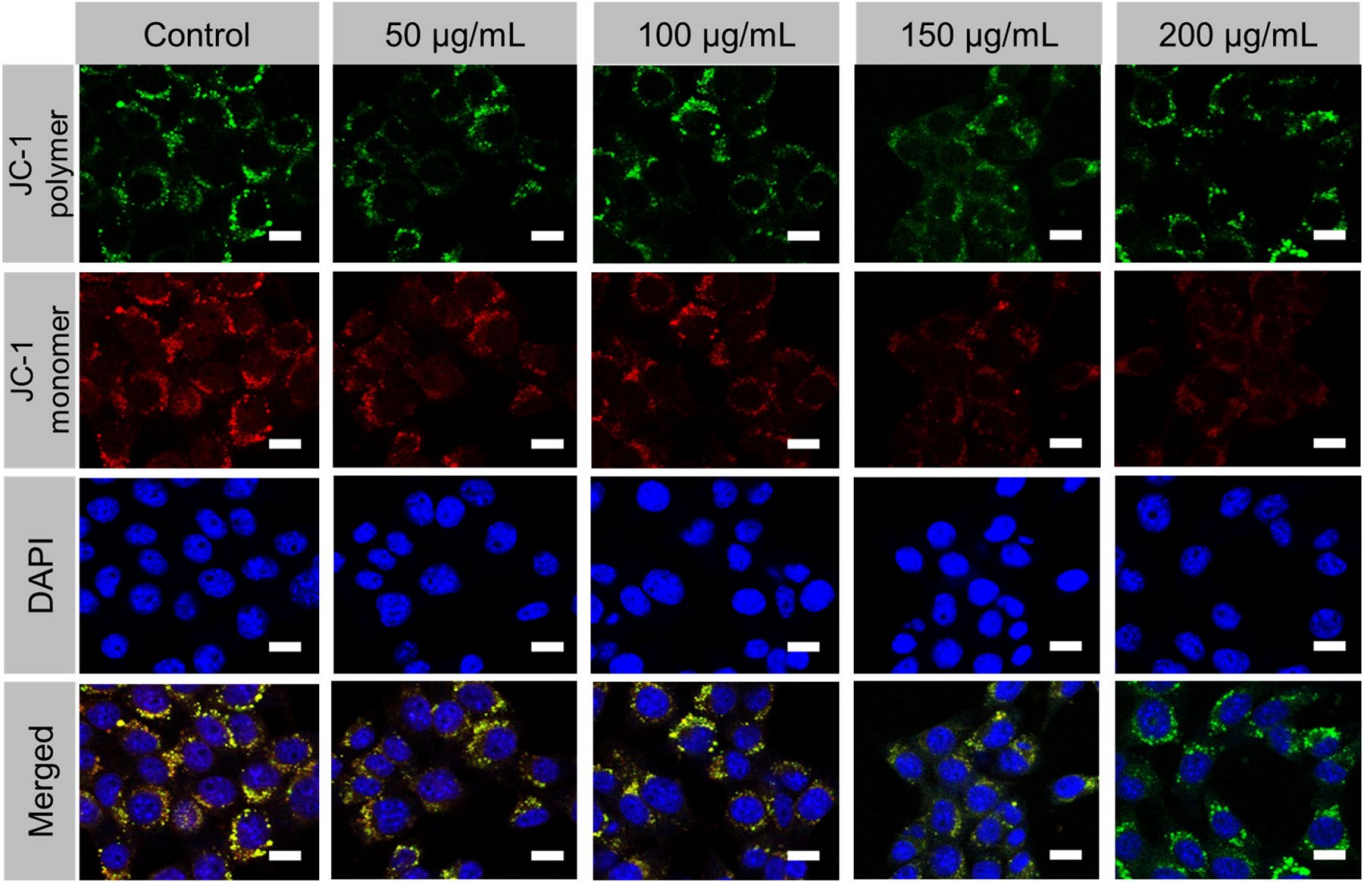
PEG@mTa2O5 triggered the ΔΨm collapse by HCMECs. The ΔΨm decline induced by PEG@mTa_2_O_5_ by HCMECs as shown by the images of ΔΨm. Scale bar 50 µm.

**Figure 7.**
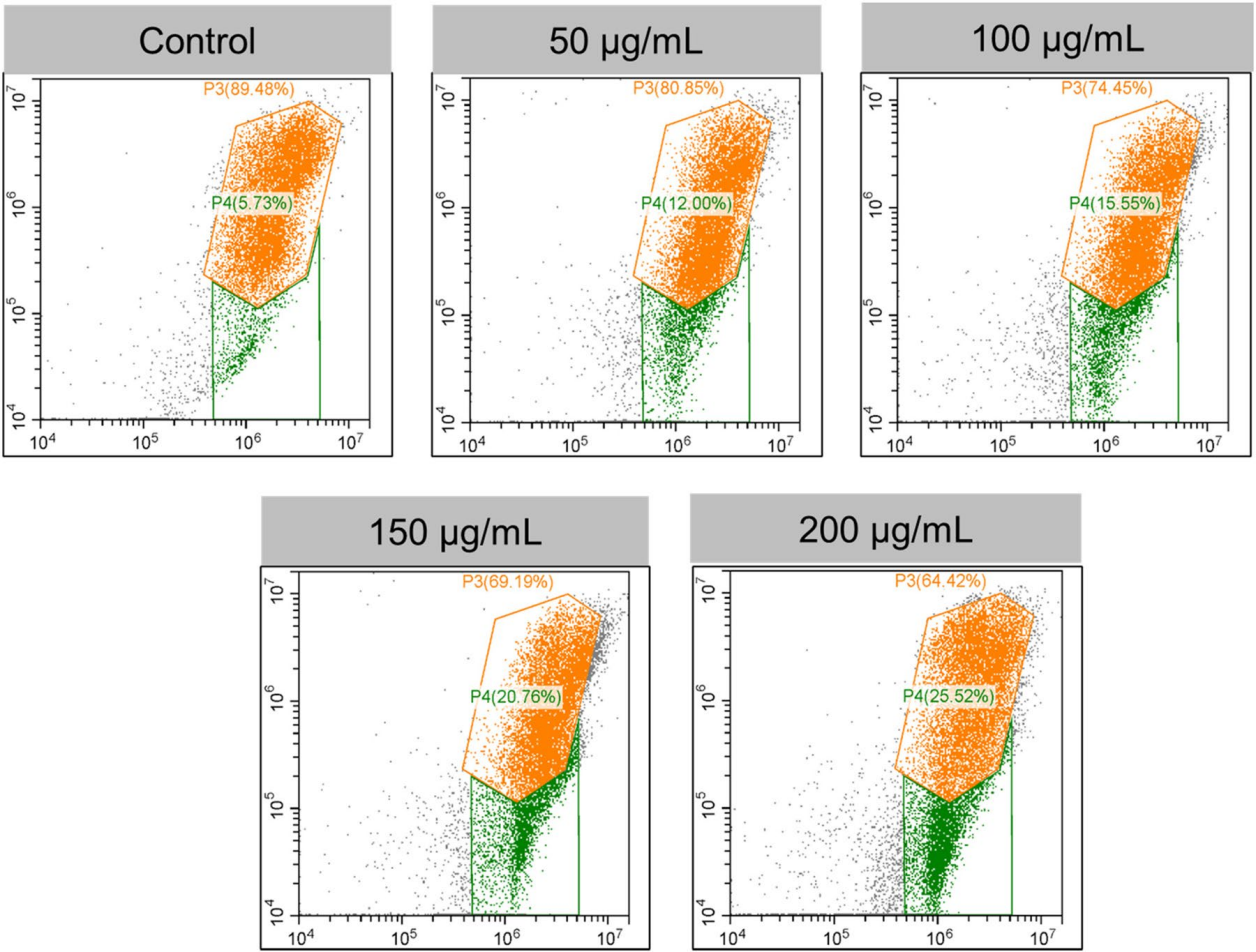
JC-1 Red-Green fluorescence intensity ratio by flow cytometer confirms the PEG@mTa_2_O_5_ by HCMECs mitochondrial membrane potential.

The ΔΨm (or MMP), an index for mitochondrial destruction, was also calculated considering the mitochondrial damage found in ultrastructure, which may be attributable to the colocalization of PEG@mTa_2_O_5_ in mitochondrial and PEG@mTa_2_O_5_-triggered ROS production (Figure 6). Endothelial cells treated with PEG@mTa_2_O_5_ showed evidence of an ΔΨm collapse, as evidenced by a reduction in red fluorescence (aggregates JC-1) and a rise in green fluorescence (monomers JC-1) (Figure 7).

### 3.7. Calcium overload induced by PEG@mTa_2_O_5_

**Figure 8.**
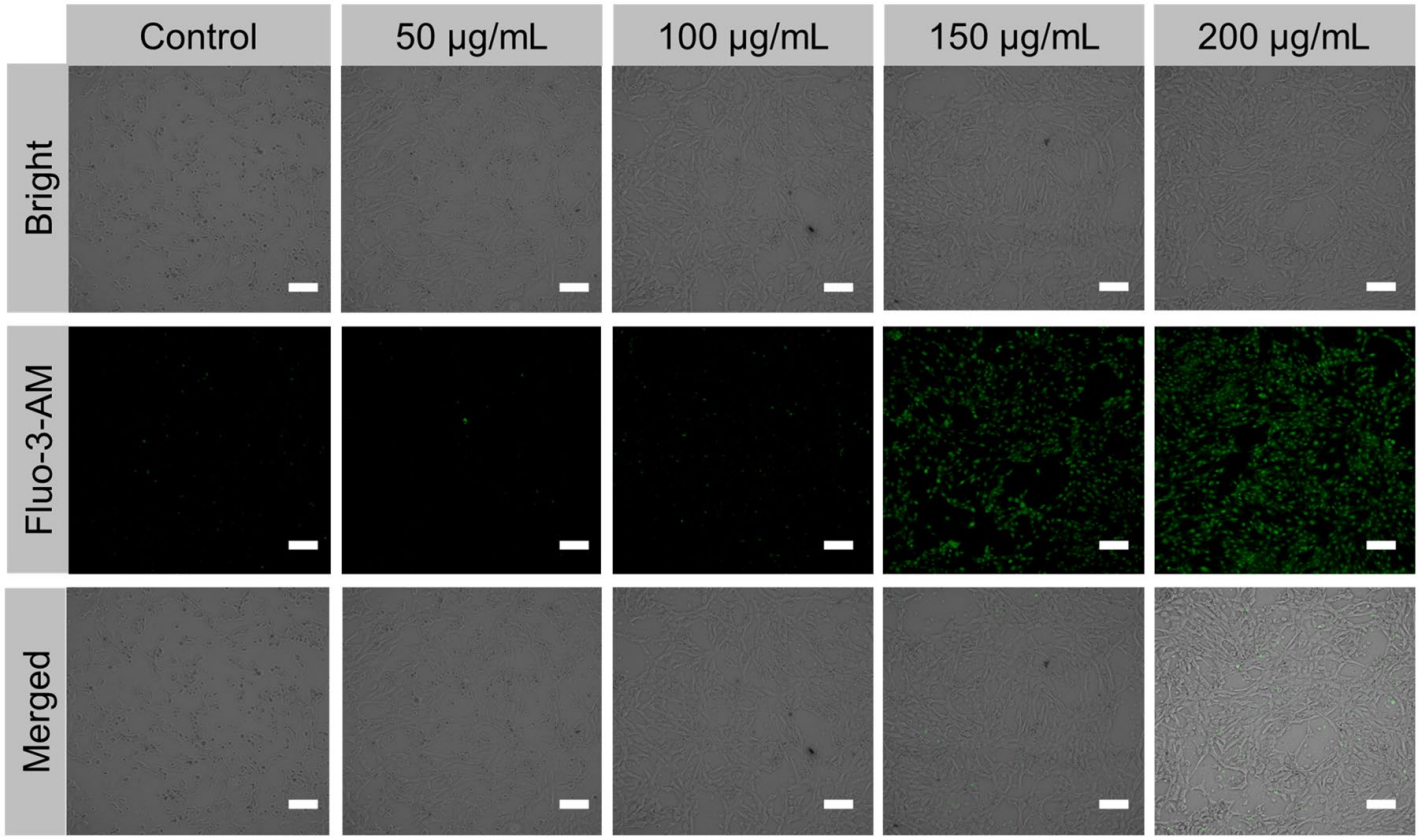
Calcium overload triggered by PEG@mTa_2_O_5_ by HCMECs. After PEG@mTa_2_O_5_ with different concentrations for 24 h, HCMECs were stained with Fluo-3 AM, a calcium indicator dye. Scale bar 100 µm.

A fluo-3 AM calcium indicator probe dye was employed for both LSCM monitoring and FCM quantitative analysis, and the results verified that PEG@mTa_2_O_5_ induced a considerable increase in intracellular Ca^2+^ (Figure 8).

### 3.8. ER stress triggered by PEG@mTa_2_O_5_

**Figure 9.**
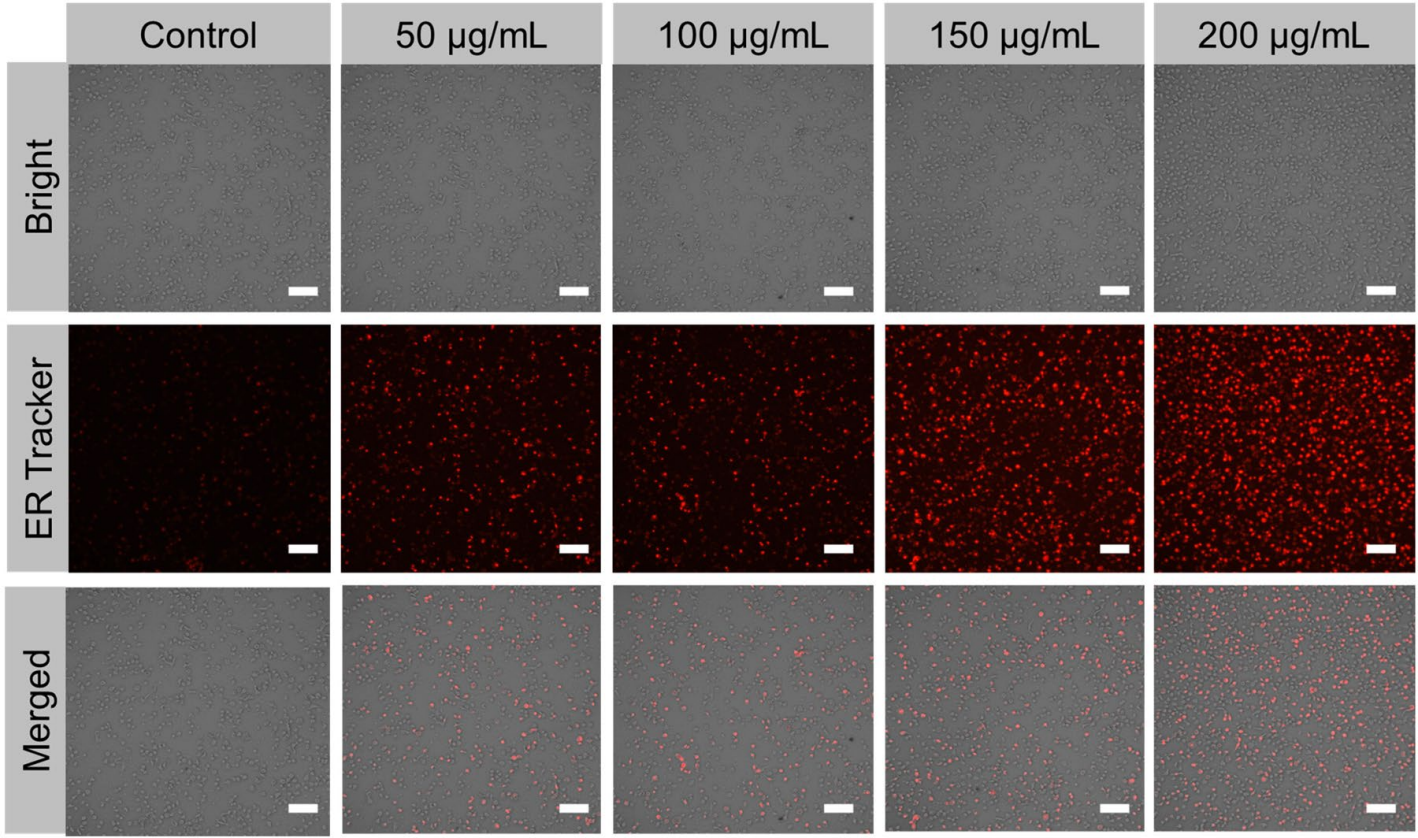
ER staining after PEG@mTa_2_O_5_ by HCMECs. After PEG@mTa_2_O_5_ treatment for 24 h, ER-dye. Scale bar 50 µm.

**Figure 10.**
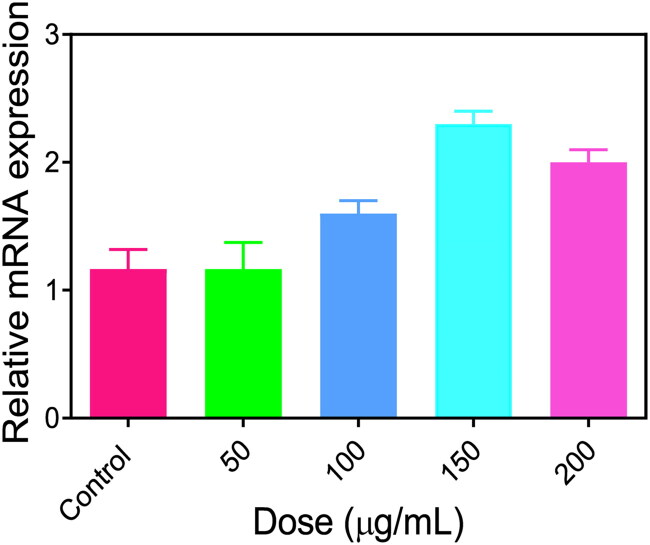
ER stress induced by PEG@mTa_2_O_5_ by HCMECs. PEG@mTa_2_O_5_ induced improved expressions of GRP78/BiP mRNA in HCMECs.

Since TEM examination revealed ER aggregates in PEG@mTa_2_O_5_-treated endothelial cells, we further investigate the potential function of ER in PEG@mTa_2_O_5_-induced endothelial death. First, confocal image analysis indicated that PEG@mTa_2_O_5_ could induce ER stress since there was a notable rise in the ER-Tracker fluorescence intensity (Figure 9). The transcriptional activation of the merging of the XBP1 and GRP78/BiP are two widely utilized indicators of the ER stress response. Specifically, PEG@mTa_2_O_5_-treated endothelium cells exhibited the XBP1 merging and enhanced GRP78/BiP mRNA expressions (Figure 10). This information corroborated previous findings that PEG@mTa_2_O_5_ causes ER stress.

### 3.9. Apoptotic pathway by PEG@mTa_2_O_5_

**Figure 11.**
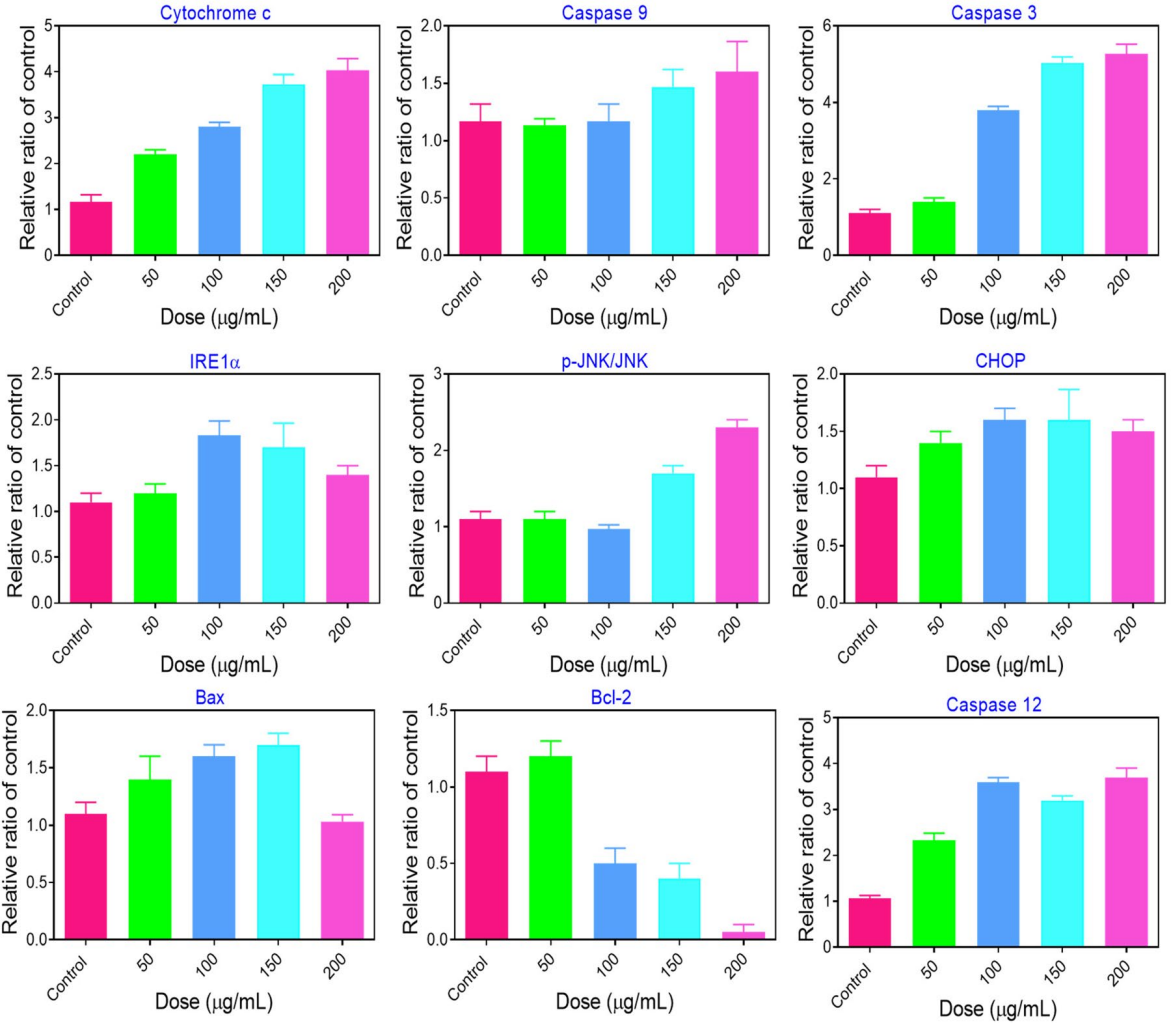
ER stress-mitochondria cascade-mediated apoptotic pathways induced by PEG@mTa_2_O_5_ in HCMECs. After PEG@mTa_2_O_5_ treatment for 24 h, the protein quantification of cytochrome c, Caspase-9, Caspase-3, IRE1a, phospho-JNK, CHOP, Bax, Bcl-2, Caspase-12.

The intrinsic mitochondria-mediated signaling pathway, considered by increased representations of Caspase-9, Caspase −3, and cytochrome c, was detected in PEG@mTa_2_O_5_-treated HCMECs, and the ER stress-related Caspase 12 and CHOP, both of which are essential for the ER stress-facilitated induction of apoptosis, were upregulated in PEG@mTa_2_O_5_-treated HCMECs (Figure 11). Remarkably, the upregulation of Bax and downregulation of Bcl-2, as well as the elevation of IRE1 and phosphorylation of JNK, all confirmed interactions involving ER stress- and mitochondria-mediated apoptosis signaling pathway (Figure 11). These findings revealed that PEG@mTa_2_O_5_-induced endothelium apoptosis was mediated by ER stress and mitochondrial caspases.

### 3.10. Systemic toxicity profile of PEG@mTa_2_O_5_

**Figure 12.**
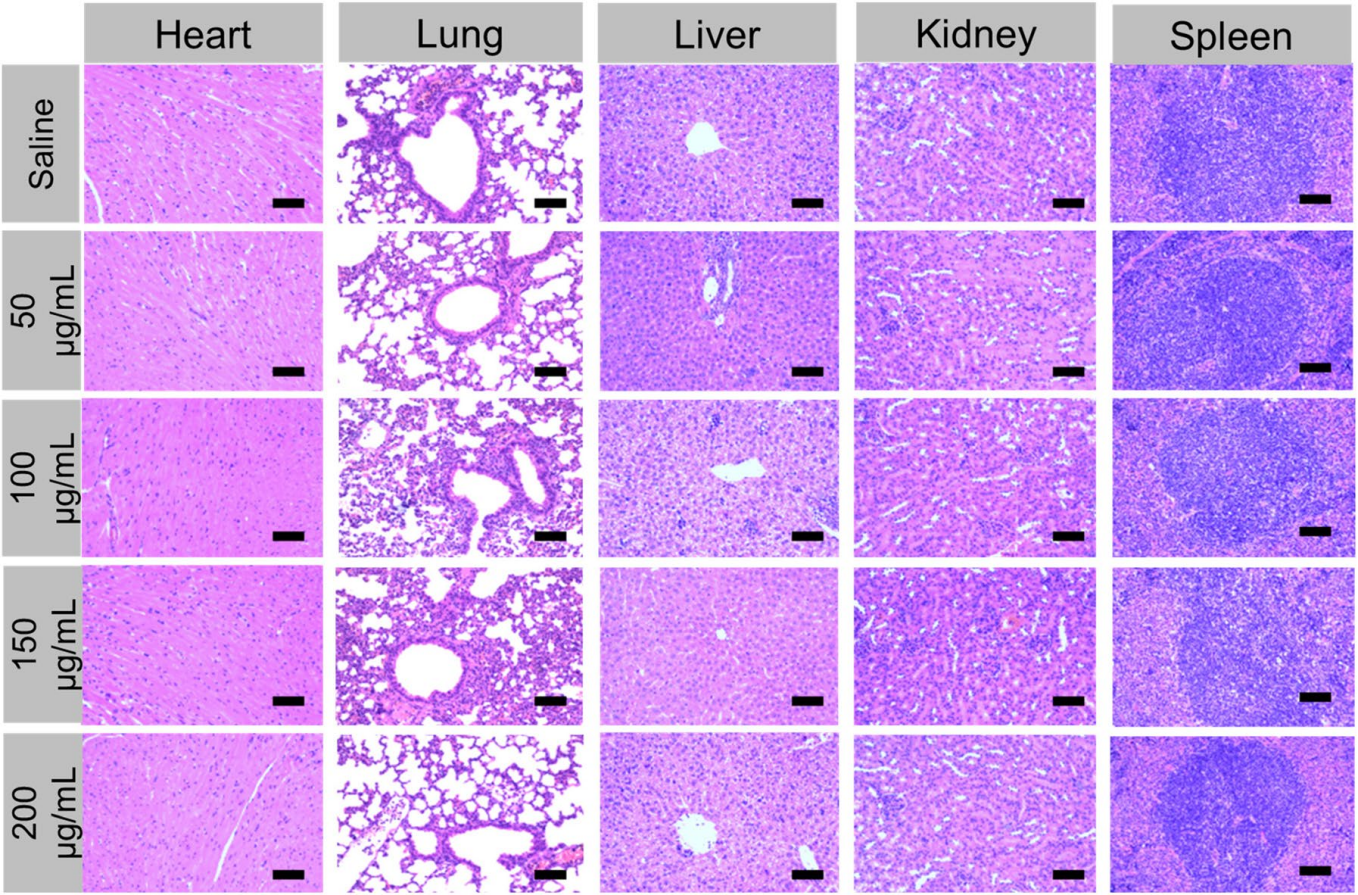
H&E staining images of major organs (heart, liver, spleen, lung, and kidney) were collected from different treatment groups on day 9 post-treatment.

**Figure 13.**
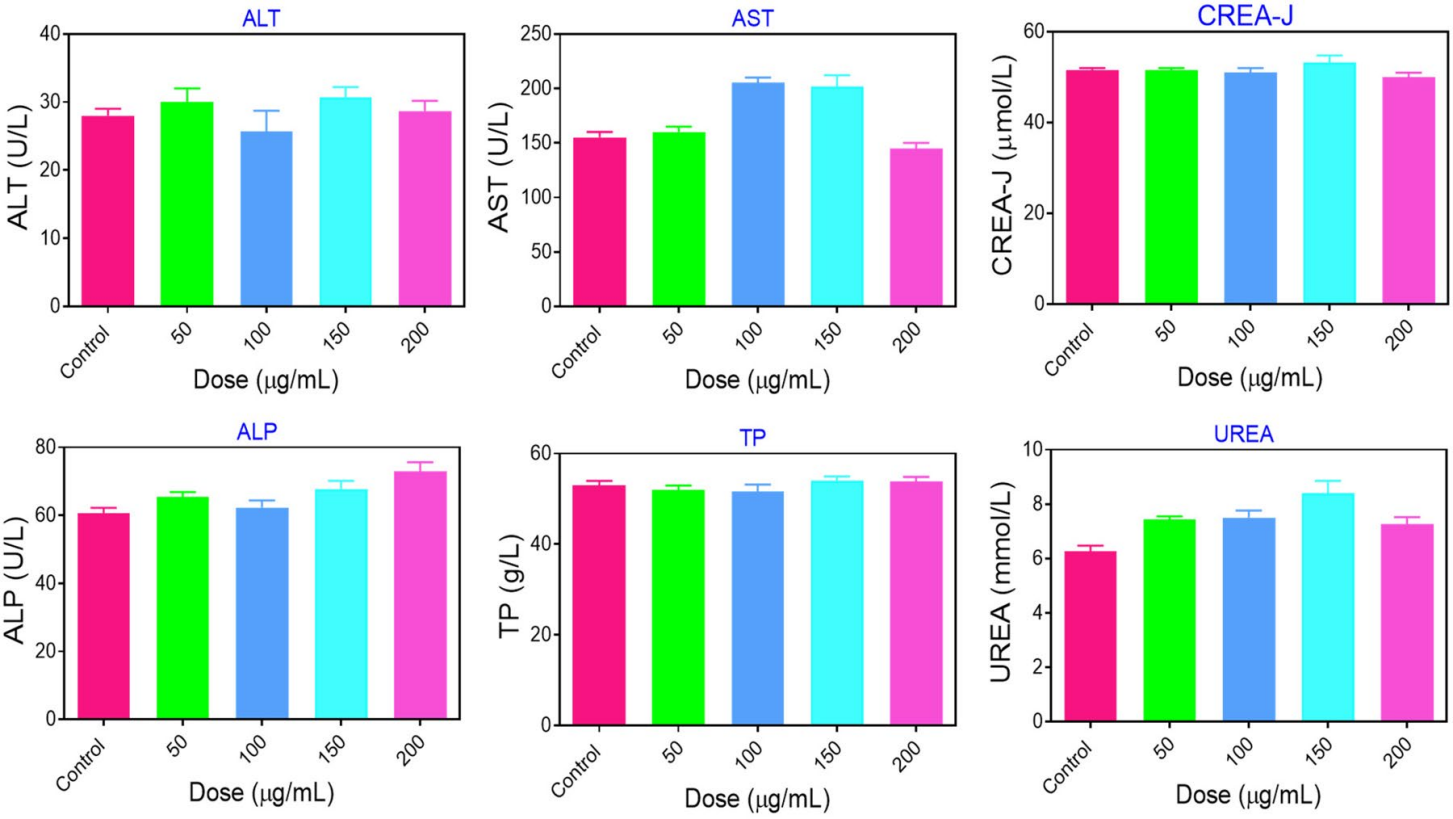
Biochemical blood parameters of PEG@mTa_2_O_5_ with different concentration.

Biosafety behavior was then examined in healthy BALB/C mice, with in vitro cytotoxicity and in vivo toxicities. After 9 days of obtaining the intravenous injection of 200 μL saline and PEG@mTa_2_O_5_ (50, 100, 150, and 200 μg/mL), standard blood and serum biochemistry tests were performed to assess in vivo hemocompatibility and liver function. As shown in Figure 12, the hematological findings of the group treated with PEG@mTa_2_O_5_ (50, 100, 150, and 200 μg/mL) showed no significant changes from the saline-injected control group. While this was happening, no apparent differences were detected in the ALT/AST ratio between the PEG@mTa_2_O_5_ (50, 100, 150, and 200 μg/mL) and saline groups, confirming that PEG@mTa_2_O_5_had no liver toxicity in mice (Figure 13). H/E-stained findings further indicate no substantial inflammation and systemic tissue damage in the major organs, with kidney, lung, spleen, liver, and heart, on the day 30^th^ after subcutaneous administration of PEG@mTa_2_O_5_ (Figure 12). This confirms PEG@mTa_2_O_5_ polymerized organosilica nature and tiny particle size, which had no detrimental effects on mice models and are incredibly beneficial for pharmacological and biomedical purposes.

## 4. Discussion

Atherosclerosis, the primary etiology of CVD, continues to be a leading cause of death worldwide (Nichols et al., 2014). Tricot et al. found that 60% of studied lesions showed endothelial apoptosis (Tricot et al., 2000). The endothelial cells underwent rapid necrotic cell death within minutes after being treated with PEG@mTa_2_O_5_, as revealed by Napierska et al. (Napierska et al., 2009). Subsequent research by Liu and Sun found that PEG@mTa_2_O_5_ significantly caused apoptosis in HCMECs at concentrations as low as 50 g/mL. In this case, the induction of apoptosis by PEG@mTa_2_O_5_ was validated both by morphological observation of apoptotic cells and by quantitative analysis of their numbers (Wang et al., 2018). Phosphatidylserine exposure, an early apoptotic indication, was readily apparent even at the lowest dosage (12.5 µg/mL, 24-h, Figure 4). Endothelial apoptosis was induced by PEG@mTa_2_O_5_ in a dose- and size-dependent fashion. Developing endothelial apoptosis after exposure to PEG@mTa_2_O_5_ may be a significant biological consequence in this setting and may harm the cardiovascular system. However, a thorough understanding of the underlying processes remains elusive (Liu & Sun, 2010).

Recent research has provided insight into the molecular pathways that play a role in the apoptotic pathway (Ulukaya et al., 2011). Endothelial damage, which contributes to the development of atherosclerosis, is known to be caused by oxidative stress. Many reactive oxygen species (ROS) come from the mitochondria. Mitosomal reactive oxygen species (ROS) generation (Figure 5) can be triggered by the internalized PEG@mTa_2_O_5_ (Figure 5). This, in turn, can cause mitochondrial damage, defined by induction of ΔΨm collapse (Figure 6). Additionally, it has been suggested that ingested PEG@mTa_2_O_5_ may interfere with mitochondrial formation by disrupting mitochondrial dynamics (fission and fusion) (Guo et al., 2018). Excessive levels of mitochondrial reactive oxygen species (ROS) will eventually affect vascular barrier function and speed up atherogenesis by causing mitochondrial dysfunction and apoptosis. In this study, we show that cytotoxic processes like apoptosis and necrosis are invariably the result of excessive mitochondrial ROS production in HCMECs (Figure 7). A dose-response release of cytochrome c increased Bax protein while downregulating Bcl-2 protein and enhanced Caspase-3, and Caspase-9 expression is a sign that mitochondrial apoptotic signaling has occurred in endothelial cells after severe mitochondrial injury (Yang et al., 2013; Shukur et al., 2016; Yang et al., 2017), just as they have in other cell types (Figure 7).

If protein folding processes are disrupted, vascular endothelial cells may experience NP-induced unfolded protein response (UPR or ER stress) (Sun et al., 2011). However, there is a dearth of information on the interplay between ER stress and PEG@mTa_2_O_5_ exposure, and the underlying processes remain unclear. According to a study by Liu et al. (Liu & Sun, 2010), PEG@mTa_2_O_5_ can cause ER perturbations in human hepatoma cells, triggering an ER stress response (Huh7). Consistent with the endoplasmic reticulum accumulation observed by the TEM image (Figure 10), HCMECs treated with PEG@mTa_2_O_5_ showed dramatically increased ER staining (Figure 10). Endothelial cells treated with PEG@mTa_2_O_5_ showed increased GRP78/BiP expression and XBP1 splicing (Figure 10). They monitored the activation of ER stress, and ER response signaling pathways may aid in restoring ER function and maintaining cellular homeostasis in the first stages of ER stress (Bernales et al., 2007; Jiang et al., 2015; Gutiérrez & Simmen, 2018). Upregulated CHOP following ER stress induces an apoptotic mode of cell death, and caspase-12 is thought to be the executor of the apoptotic process triggered by ER stress. The enhanced caspase-12 and CHOP expressions verified an ER stress facilitated cell death in PEG@mTa_2_O_5_-treated HCMECs (Figure 11). Overwhelmed ER stress may be too responsible, as it promotes cell death by increasing cell damage (Masaki et al., 2009).

ER-mitochondria crosstalk was essential for controlling metabolism and apoptosis. It has only been recently discovered that Bcl-2 family members play a role in the crosstalk between the endoplasmic reticulum (ER) and the mitochondria during apoptosis (Bhandary et al., 2012). The release of cytochrome c, caspase-3 activation, and the onset of apoptosis are all factors that may be affected by the Bcl-2/Bax ratio, as has been shown in several studies (Tabas & Ron, 2011). Bcl-2 and Bax are involved in calcium homeostasis in the ER and exist in mitochondria. Bcl-2 inhibits the release of Ca^2+^ ions into the cytosol from the endoplasmic reticulum (ER), while Bax promotes this release. In addition, the ROS produced might aid in releasing Ca^2+^ from lumen ER to the cytosol, leading to the misfolding of proteins and ER stress-mediated death (Masaki et al., 2009). Ca^2+^ absorption into mitochondria, disruptions in mitochondrial dynamics and electron transport, ΔΨm-collapse, and mitochondria-facilitated death are all potential outcomes of cytosolic Ca^2+^ excess. Here, we found that the expressions of caspase-3, caspase-9, and cytochrome c were upregulated in PEG@mTa_2_O_5_-treated HCMECs, whereas Bcl-2 and Bax were down-regulated (Figure 8), which might provide to the excess cytosolic Ca^2+^ shown in Figure 8.

It has been hypothesized that JNK mediates the transition between the adaptive and apoptotic UPRs by controlling Bax activation and Bcl-2 phosphorylation. Previous research has shown that extended ER stress causes apoptosis by activating IRE1 in the ER via the UPR or by activating downstream effectors (Malhotra & Kaufman, 2007). Apoptosis triggered by ER stress may include a cascade of events beginning with the phosphorylation of JNK and progressing to the mitochondria (Lee et al., 2011). We found that PEG@mTa_2_O_5_-treated HCMECs significantly increased IRE1 expression, which was accompanied by phosphorylation of JNK and changes in the Bcl-2 family (Figure 11). Bax and BAK, both members of the pro-apoptotic Bcl-2 family, are found together on the ER membrane and initiate apoptosis by recruiting caspase-12. We hypothesize that PEG@mTa_2_O_5_ triggered apoptosis in HCMECs via signaling ER stress-mediated caspase-12, CHOP, and IRE1/JNK to the mitochondrial through Bcl-2 (Bcl-2 and Bax). Hence, to the best of our knowledge, the current investigation results are the first to demonstrate that ER stress-mitochondria-mediated apoptosis signaling pathway was engaged in PEG@mTa_2_O_5_-triggered endothelium cell death, which in turn leads to atherosclerosis.

## 5. Conclusions

In conclusion, utilizing HCMECs, we found that PEG@mTa_2_O_5_ could be uptake and cellular organelles deposition, including ER-stress, mitochondria, and lysosome. Consequently, the PEG@mTa_2_O_5_ induced significant ER stress and mitochondrial, calcium overload, oxidative stress, and cell death. Involvement of the ER stress-mediated mitochondrial apoptotic cascade signaling in the PEG@mTa_2_O_5_-induced death of endothelial cells is remarkable. Our work underscores the need for comprehensive research on the connections among intracellular organelles in toxicological assessment. Notably, the systemic toxicity and blood compatibility profile of PEG@mTa_2_O_5_ can greatly improve successive therapeutic outcomes of NMs while reducing their adverse side effects. It provides novel insight into the specific molecular mechanism of PEG@mTa_2_O_5_-induced endothelial apoptosis.

## Data Availability

All data generated or analyzed during this study are included in this submitted article. The raw data shall be made available upon request to the corresponding author.
